# Intermittent Preventive Treatment of Malaria Provides Substantial Protection against Malaria in Children Already Protected by an Insecticide-Treated Bednet in Mali: A Randomised, Double-Blind, Placebo-Controlled Trial

**DOI:** 10.1371/journal.pmed.1000407

**Published:** 2011-02-01

**Authors:** Alassane Dicko, Abdoulbaki I. Diallo, Intimbeye Tembine, Yahia Dicko, Niawanlou Dara, Youssoufa Sidibe, Gaoussou Santara, Halimatou Diawara, Toumani Conaré, Abdoulaye Djimde, Daniel Chandramohan, Simon Cousens, Paul J. Milligan, Diadier A. Diallo, Ogobara K. Doumbo, Brian Greenwood

**Affiliations:** 1 Malaria Research and Training Centre, Faculty of Medicine Pharmacy and Dentistry, University of Bamako, Bamako, Mali; 2 Centre de Santé de Référence de Kati, Kati, Mali; 3 London School of Hygiene & Tropical Medicine, London, United Kingdom; University of Melbourne, Australia

## Abstract

**Background:**

Previous studies have shown that in areas of seasonal malaria transmission, intermittent preventive treatment of malaria in children (IPTc), targeting the transmission season, reduces the incidence of clinical malaria. However, these studies were conducted in communities with low coverage with insecticide-treated nets (ITNs). Whether IPTc provides additional protection to children sleeping under an ITN has not been established.

**Methods and Findings:**

To assess whether IPTc provides additional protection to children sleeping under an ITN, we conducted a randomised, double-blind, placebo-controlled trial of IPTc with sulphadoxine pyrimethamine (SP) plus amodiaquine (AQ) in three localities in Kati, Mali. After screening, eligible children aged 3–59 mo were given a long-lasting insecticide-treated net (LLIN) and randomised to receive three rounds of active drugs or placebos. Treatments were administered under observation at monthly intervals during the high malaria transmission season in August, September, and October 2008. Adverse events were monitored immediately after the administration of each course of IPTc and throughout the follow-up period. The primary endpoint was clinical episodes of malaria recorded through passive surveillance by study clinicians available at all times during the follow-up. Cross-sectional surveys were conducted in 150 randomly selected children weekly and in all children at the end of the malaria transmission season to assess usage of ITNs and the impact of IPTc on the prevalence of malaria, anaemia, and malnutrition. Cox regression was used to compare incidence rates between intervention and control arms. The effects of IPTc on the prevalence of malaria infection and anaemia were estimated using logistic regression. 3,065 children were screened and 3,017 (1,508 in the control and 1,509 in the intervention arm) were enrolled in the study. 1,485 children (98.5%) in the control arm and 1,481 (98.1%) in the intervention arm completed follow-up. During the intervention period, the proportion of children reported to have slept under an ITN was 99.7% in the control and 99.3% in intervention arm (*p* = 0.45). A total of 672 episodes of clinical malaria defined as fever or a history of fever and the presence of at least 5,000 asexual forms of *Plasmodium falciparum* per microlitre (incidence rate of 1.90; 95% confidence interval [CI] 1.76–2.05 episodes per person year) were observed in the control arm versus 126 (incidence rate of 0.34; 95% CI 0.29–0.41 episodes per person year) in the intervention arm, indicating a protective effect (PE) of 82% (95% CI 78%–85%) (*p*<0.001) on the primary endpoint. There were 15 episodes of severe malaria in children in the control arm compared to two in children in the intervention group giving a PE of 87% (95% CI 42%–99%) (*p* = 0.001). IPTc reduced the prevalence of malaria infection by 85% (95% CI 73%–92%) (*p*<0.001) during the intervention period and by 46% (95% CI 31%–68%) (*p*<0.001) at the end of the intervention period. The prevalence of moderate anaemia (haemoglobin [Hb] <8 g/dl) was reduced by 47% (95% CI 15%–67%) (*p*<0.007) at the end of intervention period. The frequencies of adverse events were similar between the two arms. There was no drug-related serious adverse event.

**Conclusions:**

IPTc given during the malaria transmission season provided substantial protection against clinical episodes of malaria, malaria infection, and anaemia in children using an LLIN. SP+AQ was safe and well tolerated. These findings indicate that IPTc could make a valuable contribution to malaria control in areas of seasonal malaria transmission alongside other interventions.

**Trial Registration:**

ClinicalTrials.gov NCT00738946

*Please see later in the article for the Editors' Summary*

## Abstract 2

A randomized trial reported by Alassane Dicko and colleagues shows that intermittent preventive treatment for malaria in children who are protected from mosquitoes by insecticide-treated bednets provides substantial protection from malaria.

## Editors' Summary

**Background:**

Malaria accounts for one in five of all childhood deaths in Africa and of the one million annual malarial deaths world-wide, over 75% occur in African children <5 years old infected with *Plasmodium falciparum*. Malaria also causes severe morbidity in children, such as anemia, low birth-weight, epilepsy, and neurological problems, which compromise the health and development of millions of children living in malaria endemic areas. As much of the impact of malaria on African children can be effectively prevented, significant efforts have been made in recent years to improve malaria control, such as the implementation of intermittent preventive treatment (IPT) of malaria.

IPT involves administration of antimalarial drugs at defined time intervals to individuals, regardless of whether they are known to be infected with malaria, to prevent morbidity and mortality. IPT was initially recommended for pregnant women and recently this strategy was extended to include infants (IPTi). Now, there is also intermittent preventive treatment of malaria in children (IPTc), which is designed to protect against seasonal malaria transmission including those above one year of age.

**Why Was This Study Done?:**

Large clinical trials have shown that IPTc involving the administration of two to three doses of an antimalarial drug (sulphadoxine pyrimethamine [SP] and artesunate [AS] or amodiaquine [AQ]) during the high malaria transmission season effectively reduces the incidence of malaria. However, these studies were conducted in countries where the use of insecticide-treated bednets—an intervention that provides at least 50% protection against morbidity from malaria and is the main tool used for malaria control in most of sub-Saharan Africa—was relatively low. Therefore, it is unclear whether IPTc will be as effective in children who sleep under insecticide-treated bednets as has been previously shown in communities where insecticide-treated bednet usage is low. So to determine the answer to this important question, the researchers conducted a randomized, placebo controlled trial of IPTc with SP+AQ (chosen because of the effectiveness of this combination in a pilot study) in children who slept under an insecticide-treated bednet in an area of seasonal malaria transmission in Mali.

**What Did the Researchers Do and Find?:**

The researchers enrolled 3,017 eligible children aged 3–59 months into a randomized double-blind, placebo-controlled trial during the 2008 malaria transmission season in Mali. All children were given a long-lasting insecticide-treated bednet at the start of the study with instructions to their family on the correct use of the net. Children were then randomized into two arms—1,509 were allocated to the intervention group and 1,508 to the control group—to receive three courses of IPTc with SP plus AQ or placebos given at monthly intervals during the peak malaria transmission season. The researchers monitored the incidence of malaria throughout the malaria season and also monitored the use of long-lasting insecticide-treated bednets throughout the study period. In addition, researchers conducted a cross-sectional survey in 150 randomly selected children every week and in every child enrolled in the trial 6 weeks after the last course of IPTc, to measure their temperature, height and weight, and blood hemoglobin and parasite level.

The number of children who slept under their long-lasting insecticide-treated bednet was similar in both arms. During the intervention period, the researchers observed a total of 672 episodes of clinical malaria (defined as fever or a history of fever and the presence of at least 5,000 asexual forms of *Plasmodium falciparum* per microliter) in the control arm versus 126 episodes in the intervention arm, which is an incidence rate of 1.90 episodes per person year in the control arm versus 0.34 in the interventions arm—giving a protective efficacy of 87%. IPTc reduced the prevalence of malaria infection during the intervention period by 85% and by 46% at the end of the intervention period. The prevalence of moderate anemia was also reduced (by 47%) at the end of intervention period. The frequencies of adverse events were similar between the two arms and there were no drug-related serious adverse events.

**What Do These Findings Mean?:**

The results of this study show that in peak malarial transmission season in Mali, IPTc provides substantial additional protection against episodes of clinical malaria and severe malaria in children sleeping under long-lasting insecticide-treated bednets. In addition, intermittent preventive treatment of malaria with SP plus AQ appears to be safe and well tolerated for use in children.

**Additional Information:**

Please access these websites via the online version of this summary at http://dx.doi.org/10.1371/journal.pmed.1000407.

- This topic is further discussed in two *PLoS Medicine* research articles by Konat et al. and Bojang et al., and a *PLoS Medicine* Perspective by Beeson
- Roll Back Malaria has information about malaria in children, including intervention strategies and an information sheet on insecticide-treated bednets
- UNICEF also provides comprehensive information about malaria in children
- The Intermittent Preventive Treatment in Infants Consortium (ipti) provides information on intermittent preventive treatment in infants

## Introduction

An estimated 863 million people live in sub-Saharan Africa of whom 16.2% are under 5 y of age [1]. About 300 million people live in areas where malaria transmission is highly seasonal. Malaria remains a major cause of morbidity and mortality and is estimated to cause 881,000 deaths globally per year and sub-Saharan Africa is disproportionately affected, suffering 91% of global malaria deaths with 88% occurring in children under 5 y of age [2]. Thus, in the absence of a vaccine, simple and effective control strategies are urgently needed to reduce the malaria burden in sub-Saharan Africa. Vector control, using insecticide-treated bednets (ITNs), insecticide-treated curtains, or indoor residual spraying (IRS), can reduce mortality and morbidity from malaria substantially [3], but in high transmission settings, these interventions provide only partial protection and additional control measures are needed.

Intermittent preventive treatment (IPT) is a new approach in the prevention of malaria in infants and older children. Several randomised controlled trials have demonstrated that IPT of malaria in infants (IPTi) with sulphadoxine pyrimethamine (SP) given during routine vaccinations at approximately 2, 3, and 9 mo of age, reduces the incidence of clinical malaria by 22% to 59% [4], and this strategy has been shown to be safe and cost effective. However, in many regions of Africa, the main burden of malaria falls not on infants but on older children [5]. In parts of Africa, such as much of the Sahel and sub-Sahel, where malaria transmission is very seasonal, the incidence of severe malaria currently peaks at 2 to 3 y of age. As the overall incidence of malaria decreases in Africa in response to enhanced control efforts, an effect already being seen in some countries, it can be anticipated that the mean age of cases of malaria will increase further. For these reasons, trials have been undertaken in areas of seasonal malaria transmission to determine whether IPT in children (IPTc) could be used as an effective malaria control tool in older children. In Mali, a 69% reduction in the incidence of clinical malaria was seen in children 0–5 y old when two doses of SP were given 8 wk apart during the malaria transmission season [6]. In Senegal, SP plus a single dose of artesunate (AS), administered on three occasions at monthly intervals during the peak malaria season, reduced the incidence of clinical malaria by 86% [7]. A subsequent trial of different drug regimens showed that IPT with SP and amodiaquine (AQ) was even more effective than SP+AS, providing approximately 95% protection [8]. A further study, conducted in an area of Ghana with more prolonged transmission, found that AS+AQ monthly was more effective than AS+AQ or SP alone given every 2 mo, suggesting that for drugs such as SP and AQ, monthly administration is needed to achieve effective IPTc [9]. Bednet coverage among young children was low at each of the sites where these trials were conducted and use of ITNs was very uncommon. Use of ITNs is now a favoured approach to the control of malaria in most parts of Africa and major efforts are being made to scale up their use. With international support, ITN coverage is increasing in many malaria endemic countries in sub-Saharan Africa [10] and it is expected that almost universal coverage with ITNs in high risk groups, as called for in the Global Malaria Action Plan [11], will be achieved in many malaria endemic countries. Thus, following on the initial encouraging results obtained with IPTc, an issue that needs to be addressed urgently is whether IPTc can provide significant added benefit to the protection against malaria provided by ITNs to warrant its use as a malaria control tool in areas with seasonal transmission of malaria and a high use of ITNs. It was initially planned to address this question simultaneously in each of the three countries Mali, Burkina Faso, and Ghana, using a similar design and methods. However, the site in Ghana had to be abandoned because of delays in obtaining regulatory approval for the use of SP+AQ, the drug combination chosen for the study on the basis of the results of previous trials and knowledge of the sensitivity of *Plasmodium falciparum* to these drugs in the proposed study areas. Very similar protocols were used for the studies conducted in Burkina Faso and Mali.

## Methods

The protocol of the trial (Text S1), protocol amendment (Text S5), and CONSORT checklist (Text S2) are available as supporting information.

### Objectives

The primary objective of the study was to determine the degree to which IPTc given during the malaria transmission season reduces the incidence of clinical malaria in children who sleep under a long-lasting insecticide-treated net (LLIN). Secondary objectives were determination of the impact of this strategy on severe malaria, all cause hospital admissions, anaemia, nutrition (wasting, stunting, and being underweight), malaria infection, and molecular markers of resistance to SP and AQ.

### Study Sites

The study was conducted in two rural villages, Djoliba and Siby, and the small town of Ouelessebougou situated in the district of Kati in the savannah region of Mali. Djoliba and Siby are located 40 and 30 km south west of the capital city Bamako, respectively, and Ouelessebougou is located 80 km south of Bamako. In Djoliba and Siby, community health centres are staffed with a physician and nurses. In Ouelessebougou, the community health centre is staffed by an assistant physician and nurses, but located less than 100 m from a district health centre staffed by four physicians and six nurses. A research team composed of physicians and medical residents was established in each of the three sites to follow up and provide health care to the study participants.

Malaria transmission in the study area is highly seasonal and 80%–90% of malaria cases occur between August and November. The entomological inoculation rate (EIR) was 9.4 and 6.6 infective bites per person per season, respectively, in Siby and in Ouelessebougou, two localities far from any river and 37.3 infective bites per person per season in Djoliba located on the bank of the Niger River (Text S3). The coverage of ITNs at baseline was 33.4% (312/935) in Siby, 84.7% (563/665) in Djoliba, and 89.8% (2,207/2,458) in Ouelessebougou.

### Study Design and Participants

The study was designed as an individually randomised, placebo-controlled trial of IPTc with SP+AQ in children who received a LLIN**.** Children aged 3–59 mo were enumerated and given a census identification number including a house number to facilitate their identification at screening, enrolment, and follow-up. Recruitment was started in Djoliba followed by Siby. In these communities all available children in the target age group who were not selected for the baseline survey of drug resistance were screened and enrolled if they met the inclusion criteria. In the larger community of Ouelessebougou, children were screened for enrolment on a first-come first-served basis until the required sample size was met. Children were eligible to join the study if they were aged 3–59 mo at the time of enrolment and permanent residents of the study area with no intention of leaving during the study period. Exclusion criteria were the presence of a severe, chronic illness, such as severe malnutrition or AIDS, and a history of a significant adverse reaction to SP or AQ. Cases of an acute illness, such as malaria, were not excluded. Such cases were treated appropriately and the child randomised and retained in the trial.

### Ethics

The study protocol was reviewed and approved by the Ethical Committee of the Faculty of Medicine, Pharmacy and Dentistry, University of Bamako, Mali and by the Ethics Committee of the London School of Hygiene and Tropical Medicine. Community consent was obtained at meetings with leaders, heads of families, and other community members of each locality prior to the start of the study. Individual, written, informed consent was obtained from a parent or guardian of each child prior to screening and enrolment. A Data and Safety Monitoring Board (DSMB) was established and monitored the trial with the support of a local medical safety monitor. Current good clinical practices (cGCP) monitoring of the trial was performed by PharmaClin (http://www.pharmaclin.com).

### Interventions

Every child who was screened was provided with a LLIN (Permanet, Vestergaard Frandsen) that was marked with the child's identification number regardless of whether or not the child was enrolled. Instructions were given to the parent or guardian on how to use the net and the importance of using the net regularly was emphasized. Monitoring of utilisation of ITNs by study participants was made in 150 randomly selected children each week and in all study children during the cross-sectional survey conducted at the end of the malaria transmission season.

Eligible children were treated with a course of SP+AQ or matching placebos on three occasions at monthly intervals during the malaria transmission season, starting in August 2008. SP and AQ were manufactured by Kinapharma Limited and quality control checks on the drugs for solubility and content were performed at the London School of Tropical Medicine and Hygiene, prior to their use in the trial. Tablets met internal standards for drug solubility and content. Doses of SP and AQ were based on weight with children stratified into one of the three weight categories (5–9 kg, 10–18 kg, and ≥19 kg). SP was given at a dose of 175/8.75 mg to children 5–9 kg, 350/17.5 mg to children 10–18 kg, and 550/26.25 mg to those who weighed ≥19 kg. The corresponding doses for AQ were 70 mg, 140 mg, and 220 mg, respectively. AQ was given over 3 d. Drugs were prepackaged to facilitate administration and put in envelopes with colour codes, one for each weight group. Within each weight stratum, children were individually randomised using a computer-generated random number sequence and blocks of varying length. Treatment allocations were provided within sealed, opaque envelopes.

Drugs were given under direct observation at a research clinic by study staff. Children were observed for 30 min after drug administration. If vomiting occurred during this 30-min period, drugs were readministered. If vomiting occurred on a second occasion, this was noted but the drugs were not given again. Such children were not excluded from the trial and they were eligible to receive drugs on the subsequent 2 d and during subsequent monthly IPT rounds. If a child missed the day set for treatment, a home visit was made to enquire why the child had not been brought for treatment and the reason was recorded. If the family wished to continue with treatment but was unable to attend on the specified day, then treatment was reoffered within an interval of 7 d of the designated date. Children with an acute malaria episode were treated with artemether-lumefantrine (AL) and did not receive IPT with SP+AQ if the treatment for acute malaria was received within 7 d of the scheduled date of IPT. Such children were eligible for treatment in future treatment rounds

### Outcomes

The primary endpoint of the study was the incidence of clinical malaria; this was defined as the presence of fever (axillary temperature ≥37.5°C) or a history of fever in the past 24 h and the presence of *P. falciparum* asexual parasitaemia at a density greater or equal to 5,000 parasites per microlitre. Secondary endpoints were: (i) the incidence of clinical malaria defined as the presence of fever or a history of fever in the past 24 h and the presence of *P. falciparum* asexual parasitaemia at any density; (ii) incidence of severe malaria defined according to the WHO criteria [12]; (iii) malaria infection defined as the presence of asexual parasitaemia; (iv) mild, moderate, or severe anaemia defined as an haemoglobin (Hb) concentration <11 g/dl, <8 g/dl, and <5 g/dl, respectively; (v) hospital admission defined as a stay of at least 24 h in hospital for treatment; (vi) anthropometric indicators including wasting, stunting, and being underweight as defined by WHO [13]; and (vii) safety and tolerability measured by the occurrence of nonserious and serious adverse events.

Passive surveillance for clinical malaria started at the time of the administration of the first dose of IPTc in August 2008 and continued until the end of the malaria transmission season in November/December 2008, 6–7 wk after the last round of IPTc. Parents were encouraged to bring their child to a study health centre, where medical staff were available 24 h a day and 7 d a week, if the child became unwell. A finger prick blood sample was be obtained from all study children with fever (an axillary temperature of 37.5°C or higher) or a history of fever within the previous 24 h for preparation of a blood film, measurement of Hb concentration, and for a rapid diagnostic test (RDT) OPTIMAL_IT (Diamed AG) for malaria. Children who had a positive RDT for malaria were treated immediately with AL. Severe cases were admitted to the health centre or referred to the paediatric ward of the Gabriel Touré Hospital in Bamako. Causes of death were assessed within a month of death using a modified version of the INDEPTH post mortem questionnaire (http://www.indepth-network.org/index.php?option=com_content&task=view&id=95&Itemid=183).

Use of a LLIN was assessed by asking if a child had slept under an LLIN the previous night and the presence of the net was checked by field staff. During these home visits, the axillary temperature of each child was taken and a blood film obtained regardless of whether or not the child had fever. A RDT was performed if a child had measured fever or a history of fever within the previous 24 h and if this was positive, treatment with AL was given according to national guidelines. At the end of the malaria transmission season, a cross-sectional survey was undertaken at which every child was examined, their height and weight recorded, and a finger prick blood sample obtained for determination of Hb concentration, preparation of blood films, and collection of a filter paper sample for subsequent molecular studies. Safety and tolerability of SP and AQ were monitored passively during the study period in all the children and actively in a subset at the time of the administration of IPT (days 0, 1, and 2) and 1 d after the last dose of treatment (day 3) at each round.

### Assessment of Molecular Markers of Drug Resistance

Monitoring of the frequency of molecular markers of resistance to sulphadoxine, pyrimethamine, and AQ was performed in two cross-sectional surveys, the first at baseline in August 2008 and the second during the survey undertaken at the end of malaria transmission season. The baseline survey was conducted in 256 children randomly selected from the screening list. These children were not enrolled in the placebo control trial. Participants enrolled in the placebo control trial were surveyed about 6 wk after the third course of IPTc, at the end of malaria transmission season, to assess whether administration of IPT with SP+AQ had lead to an increase in molecular markers of resistance to these drugs. Thick and thin blood smears and blood blotted onto filter papers were collected during both surveys for molecular analysis as described below.

### Laboratory Methods

Thick blood films were air dried, stained with Giemsa, and examined for malaria parasites by two well-trained technicians. 100 high power fields were counted before a film was declared negative. Parasite density was determined by counting the number of parasites present per white blood cell (WBC) on a thick smear and assuming a WBC count of 8,000 per µl. In the case of a discrepancy (positive/negative or a difference in parasite density greater than 30%), a third reading was done. The median parasite density of two or three readings was used. An external quality control of slide reading performed by the Malaria Diagnosis Centre of Excellence (MDCoE) of the Walter Reed/Kenya Medical Research Institute, in Kisumu, Kenya, showed an overall concordance of more than 90% on parasite detection and 100% on species identification (Text S4). Hb concentrations were measured using a haemoglobin analyzer (Hemocue HB 301) on blood obtained by finger prick.

Filter paper samples from children with a mono-infection of *P. falciparum* on blood smears were analysed by nested PCR for mutations at codons 51, 59, and 108 of the *dhfr* gene, 437 and 540 of the *dhps* gene, 76 of mutations in the *P. falciparum* chloroquine transporter gene (*pfcrt*), and 86 of the *P. falciparum* multidrug resistance gene one (*pfmdr1*) according to published methods [14]–[16]. Cases of mixed infection (wild type and mutant) were categorized as mutant.

### Sample Size

Calculation of sample size was based on the assumptions that the clinical attack rate measured by passive surveillance would be 1.0–2.0 attacks per child per year in unprotected children aged 3–59 mo living in the study areas and that sleeping under an LLIN would reduce this attack rate by half to 0.5 to 1.0 clinical episode per child per year. Assuming that children experienced an average of 0.5 clinical episodes per child per year of sufficient severity to present to a health facility, to detect a 20% reduction in this incidence (i.e., from 0.5 to 0.4 attacks per child per year) in children who receive IPTc, the smallest reduction that would be likely to make IPTc a worthwhile investment, and allowing for a 20% loss to follow-up, we estimated that approximately 2,000 children (1,000 in each arm) were required for a study with 90% power at the two-sided 5% level of significance [17]. After the site in Ghana was dropped, the sample size was increased to 1,500 participants per arm, after an amendment was made to the protocol (Text S5), which would have 80% power to detect a two-thirds reduction in the incidence of severe malaria, assuming an incidence of 2% in children in the control arm. The study was not powered to detect a smaller reduction in the incidence of severe malaria but the analysis plan included provision for combination of the results of this trial with those of a parallel study conducted in Burkina Faso to provide sufficient size to allow detection of a smaller impact of IPTc on this end point.

### Data Management and Analysis

Data were collected on standardized forms, double-entered, and verified using MS Access and then exported to Stata (StataCorp) for additional cleaning and analysis. A data analysis plan was written and submitted to the DSMB prior to analysis. The final, cleaned database was locked and a copy sent to the DSMB. An intention-to-treat analysis was performed. Incidence rates of clinical malaria, severe malaria, and hospital admissions were calculated by dividing the number of episodes by the total child days at risk. Children were not considered at risk for 21 d after each type of a malaria episode and these days were not included in the calculation of the child days at risk. The incidence rates in the two treatment groups were compared using Cox regression to estimate the incidence rate ratio, with adjustment for age, gender, and locality, and using a robust standard error to allow for the lack of independence among repeated episodes in the same child. The protective effect (PE) of IPTc was computed as 1 minus the incidence rate ratio. Time to first episode of clinical malaria in the two arms was examined using Kaplan-Meier plots and compared using log rank test. Anthropometric data at enrollment and at the end of season cross-sectional survey were converted into weight-for-age, height-for-age, and weight-for-height *z*-scores using WHO's anthropometric software (www.who.int/childgrowth/software/en). Underweight, stunting, and wasting were defined as *z*-scores of <−2 for the relevant indicator [13]. Changes in weight and height between the two groups were compared using Student's *t* test. Frequencies of single mutations as well as the triple mutant (*dhfr* 51+59+108) and quadruple mutant (triple mutant + *dhps* 437) genotypes were determined and compared between treatment arms and between the beginning and end of the study. Proportions of children with binary outcomes were compared between the two groups using Pearson's Chi square test or generalized linear models adjusted for age, gender, and locality.

## Results

### Trial Profile and Baseline Data

**Figure 1:**
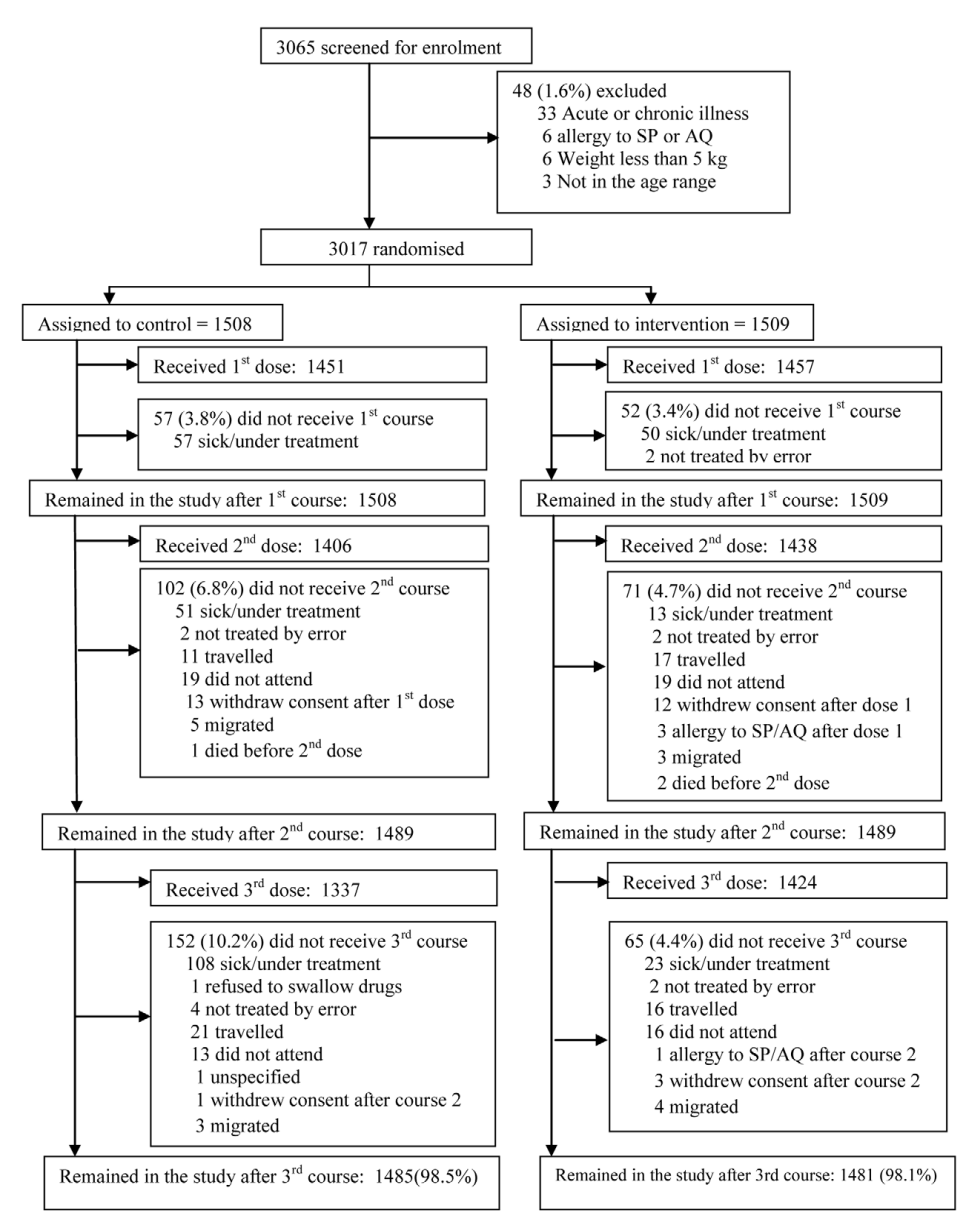
Trial profile.

The trial profile is summarised in Figure 1. A total of 3,065 children were screened of whom 3,017 (1,509 in the IPTc arm and 1,508 in the placebo arm) (98%) were enrolled. Reasons for exclusion are shown in Figure 1. The proportion of children who completed the follow-up to day 42 after the last round of IPTc was similar in the control and in the intervention arms (98.5% and 98.1%, respectively). The reasons for withdrawal were withdrawal of consent (*n* = 29), migration to another location (*n* = 15), a history of allergy to study drugs (*n* = 4 with two cases confirmed), and death (*n* = 3). There were no significant differences between intervention and control groups with regard to their age and gender distribution, nor in the prevalence of fever, wasting, or stunting at the time of enrolment (Table 1).

**Table 1:** Baseline characteristics of enrolled children at the time of administration of the first dose of IPTc.

Characteristics	IPTc	Placebo
	Percent (*n/N*)	Percent (*n/N*)
***Age (mo)***		
3–11	18.2 (274/1,509)	18.5 (278/1,508)
12–23	22.5 (339/1,509)	20.5 (309/1,508)
24–35	20.5 (310/1,509)	22.0 (**3**32/1,508)
36–47	20.0 (302/1,509)	19.4 (293/1,508)
48–59	18.8 (284/1,509)	19.6 (296/1,508)
***Gender***		
Male	47.7 (720/1,509)	50.1 (755/1,508)
Female	52.3 (789/1,509)	49.9 (753/1,508)
***Weight (kg)***		
5–9	34.8 (525/1,509)	34.7 (523/1,508)
10–8	63.1 (952/1,509)	63.2 (953/1,508)
≥19	2.1 (32/1,509)	2.1 (32/1,508)
***Nutritional factors***		
Underweight	16.1 (238/1,480)	15.1 (223/1,477)
Wasting	11.0 (163/1,480)	12.5 (185/1,477)
Stunting	22.7 (336/1,480)	23.8 (352/1,477)
***Fever***	7.2 (105/1,460)	7.6 (111/1,464)

### LLIN Usage

Usage of LLINs was assessed for 590 children in the control group and for 591 children in the intervention group during weekly home visits, undertaken without prior warning, during the course of the intervention period. Usage of an LLIN was high in each of the three study localities and similar between the two groups (99.7% in the control group versus 99.3% in the intervention arm; *p* = 0.45).

### The Impact of IPTc on Malaria

**Figure 2:**
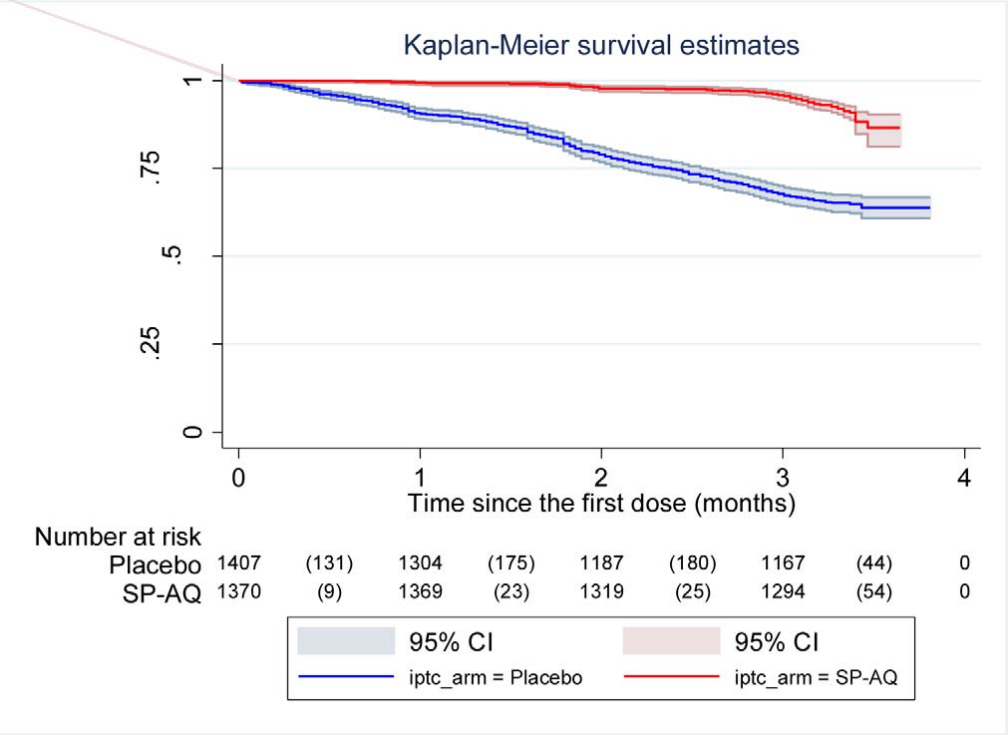
Time to first episode of clinical malaria defined as fever (temperature ≥37.5°C) or history of fever in the last 24 h and parasitaemia ≥5,000/µl in the intervention and control arms. Kaplan-Meier survival estimates with pointwise 95% confidence bands.

Among children with fever or history of fever who had an RDT positive result, 8.8% (112/1,277) turned out to have negative parasitaemia after microscopical diagnosis of malaria. The impact of IPTc on episodes of malaria detected through passive surveillance is presented in Table 2. The incidence of episodes of uncomplicated malaria (fever or a history of fever in the last 24 h and asexual parasitaemia ≥5,000/µl) was much lower among children in the IPTc arm than among those in the control arm (0.34 episodes per child/year versus 1.9 episodes per child/year). The PE against malaria adjusted for age, gender, and location was 82% (95% confidence interval [CI] 78%–85%) (*p<*0.001). An analysis of time to the first episode of clinical malaria, defined as above, also indicated a strong protective effect of IPTc (*p*<0.001) (Figure 2). The incidence of malaria defined as fever or a history of fever in the last 24 h and positive asexual parasitaemia of any density was also much lower in children in the IPTc arm compared to those in the control arm (0.41 episodes per child/year versus 2.4 episodes per child/year), giving a protective efficacy of 83% (95% CI 80%–86%) (*p*<0.001). Only 17 cases of severe malaria occurred during the follow-up period, 15 in the control group, and two in the intervention group (Table 2), giving a protective efficacy of 87% (95% CI 42%–99%) (*p* = 0.001). The two cases of severe malaria in the intervention arm, one of whom died, occurred more than 3 wk after the third course of IPT.

**Table 2:** Impact of IPTc on episodes of clinical malaria in children in Mali.

Outcomes	IPTc			Placebo			Unadjusted IRRs (95% CI)	*p*-Value	Adjusted^c^ IRRs (95% CI)	PE (95% CI)	*p*-Value
	*n* Episodes	Years at Risk^a^	Incidence Rate (95% CI)^b^	*n* Episodes	Years at Risk	Incidence Rate (95% CI)^b^					
Fever or history of fever and any asexual parasitaemia	149	362.15	0.41 (0.35–0.48)	832	345.64	2.40 (2.25–2.58)	0.17 (0.14–0.20)	<0.001	0.17 (0.14–0.20)	83 (80–86)	<0.001
Fever or history of fever and parasitaemia ≥5,000	126	369.41	0.34 (0.29–0.41)	672	354.14	1.90 (1.76–2.05)	0.18 (0.15–0.22)	<0.001	0.18 (0.15–0.22)	82 (78–85)	<0.001
Severe malaria	2	399.10	0.005 (0.0006–0.0181)	15	400.87	0.037 (0.0209–0.0617)	0.13 (0.01–0.58)	0.001	—	87 (42– 99)	0.001

a Children were not considered at risk for 21 d after each type of a malaria episode.

b Incidence rate/child/year. Note the incidence relate refers to only the 3-mo surveillance period and is not an annual rate.

c Adjusted for age, gender, and location. 95% CI constructed using a robust standard error.

IRR, incidence rate ratio.

Incidence rates and the PE of IPTc against clinical malaria by locality and age category are presented in Table 3. Although the incidence of clinical malaria varied substantially between the three study localities, the PE of IPTc was similar in all three areas regardless of the definition of clinical malaria used. PE was higher in the lower age groups (3–11 mo and 12–23 mo) compared to the older age groups (≥24 mo) when the definition of clinical malaria that incorporated the presence of parasitaemia ≥5,000/µl or any parasitaemia was used (test for effect modification *p*≤0.001 and *p* = 0.003, respectively).

**Table 3:** Effect of area of residence and age on the protective efficacy of IPTc against clinical episodes of malaria.

Outcomes According to Area of Residence and Age Category	IPTc		Placebo		Unadjusted RR (95% CI)	*p*-Value	Adjusted RR (95% CI)	PE (95% CI)	*p*-Value
	Episodes (Years at Risk)	Incidence Rate^a^	Episodes (Years at Risk)	Incidence Rate^a^					
**Clinical malaria defined as fever or history of fever in the last 24 h and asexual parasitaemia ≥5,000/µl**									
***Locality***									
Djoliba	11 (73.77)	0.15 (0.08–0.27)	74 (73.49)	1.00 (0.80–1.26)	0.13 (0.10–0.18)	<0.001	0.15 (0.08–0.28)	85 (72–92)	<0.001
Siby	70 (93.32)	0.75 (0.59–0.94)	308 (90.57)	3.40 (3.04–3.80)	0.13 (0.07–0.24)	<0.001	0.22 (0.17–0.29)	78 (71–83)	<0.001
Ouelessebougou	45 (202.55)	0.22 (0.17–0.30)	292 (190.20)	1.53 (1.37–1.72)	0.21 (0.16–0.26)	<0.001	0.14 (0.10–0.20)	86 (80–90)	<0.001
***Age (mo)***									
3–11	6 (68.64)	0.09 (0.04–0.19)	52 (66.60)	0.78 (0.59–1.02)	0.11 (0.05–0.26)	<0.001	0.13 (0.05–0.29)	87 (71–95)	<0.001
12–23	12 (83.00)	0.14 (0.08–0.25)	134 (72.40)	1.85 (1.56–2.19)	0.08 (0.04–0.14)	<0.001	0.07 (0.04–0.13)	93 (87–96)	<0.001
24–35	38 (76.63)	0.50 (0.36–0.68)	173 (77.96)	2.22 (1.91–2.58)	0.22 (0.15–0.33)	<0.001	0.23 (0.15–0.34)	77 (66–85)	<0.001
36–47	36 (72.51)	0.50 (0.36–0.69)	153 (67.84)	2.26 (1.92–2.64)	0.22 (0.15–0.31)	<0.001	0.21 (0.15–0.31)	79 (69–85)	<0.001
48–59	34 (66.91)	0.51 (0.36–0.71)	156 (66.43)	2.34 (2.00–2.74)	0.21 (0.14–0.32)	<0.001	0.22 (0.15–0.32)	78 (68–85)	<0.001
**Clinical malaria defined fever or history of fever in the last 24 h and asexual parasitaemia regardless of the density**									
***Locality***									
Djoliba	12 (72.05)	0.17 (0.09–0.29)	90 (73.40)	1.22 (1.0–1.50)	0.13 (0.10–0.18)	<0.001	0.14 (0.10–0.18)	86 (82 –90)	<0.001
Siby	83 (90.86)	0.91 (0.74–1.13)	372 (86.41)	4.30 (3.88–4.76)	0.13 (0.07–0.24)	<0.001	0.14 (0.07–0.26)	86 (74 –93)	<0.001
Ouelessebougou	54 (199.25)	0.27 (0.21–0.35)	370 (185.82)	1.99 (1.80–2.20)	0.21 (0.16–0.26)	<0.001	0.21 (0.16–0.26)	79 (74–84)	<0.001
***Age (mo)***									
3–11	10 (68.15)	0.15 (0.08–0.27)	72 (65.96)	1.09 (0.86–1.38)	0.13 (0.07–0.26)	<0.001	0.14 (0.07–0.27)	86 (73–93)	<0.001
12–23	15 (81.60)	0.18 (0.11–0.30)	153 (71.07)	2.15 (1.83–2.52)	0.08 (0.05–0.14)	<0.001	0.08 (0.05–0.14)	92 (86–95)	<0.001
24–35	47 (75.18)	0.62 (0.47–0.83)	206 (76.20)	2.70 (2.36–3.10)	0.23 (0.17–0.31)	<0.001	0.23 (0.16–0.33)	77 (67–84)	<0.001
36–47	39 (70.23)	0.55 (0.40–0.76)	200 (65.44)	3.06 (2.66–3.51)	0.18 (0.13–0.25)	<0.001	0.18 (0.13–0.25)	82 (75–87)	<0.001
48–59	38 (65.04)	0.58 (0.42–0.80)	194 (63.98)	3.03 (2.63–3.49)	0.19 (0.13–0.27)	<0.001	0.19 (0.13–0.28)	81 (72–87)	<0.001

a Incidence rate expressed as number of episodes/child/year. Note that this is based on the 3-mo surveillance period and does not correspond to an annual rate.

The percentage of children with malaria infection detected at weekly active surveillance visits was 13.2% (74/563) in the control group compared to 1.9% (11/575) in the intervention group, giving a protective efficacy of 85%, (95% CI 73%–92%) (*p*<0.001). At the end of the transmission season, 13.2% (188/1,423) of children in the control group were parasitaemic compared to 7.2% (101/1,405) in the intervention group, giving a protective efficacy of 46% (95% CI 31%–68%) (*p*<0.001).

### The Impact of IPTc on Anaemia

At the end of the malaria transmission season, the proportion of the children with anaemia (Hb <11 g/dl), was significantly higher in the control group compared to the intervention group (61.1% [875/1,433] versus 53.9% [766/1,422]) (PE = 12%; 95% CI 3%–20%) (*p*<0.001). The relative difference was larger for moderate anaemia (Hb <8 g/dl) with a prevalence of 3.5% (50/1,433) versus 1.9% (27/1,422) in the control and intervention groups, respectively (PE = 47%; 95% CI 15%–67%) (*p* = 0.007). No cases of severe anaemia (Hb <5 g/dl) were observed in either treatment group at the time of the postintervention survey. However, during the follow-up period, a total of eight cases of severe anaemia occurred, two in the intervention arm and six in the control arm. The two participants in the intervention group who developed severe anaemia had not received a complete course of IPT at the time that they developed their severe anaemia.

### The Impact of IPTc on Nutritional Indicators

The impact of IPTc on nutritional indicators is presented in Table 4. The proportions of children with wasting, stunting, and being underweight at the end of the malaria transmission season were similar between the control and intervention arms However, weight gain during the intervention period was 97 g (95% CI 37 g–157 g) more among children in the intervention arm compared to that recorded among children in the control arm. Changes in height were similar between the two arms with an increase of 2.3 cm (95% CI 2.2 cm–2.5 cm) in children in the intervention arm compared to an increase of 2.4 cm (95% CI 2.2 cm–2.5 cm) in children in the control arm.

**Table 4:** Effect of IPTc on nutritional indicators in children at the end of the malaria transmission season.

Nutritional Indicators	Placebo		IPTc		Adjusted Analysis	
	Percent	*n*	Percent	*n*	OR (95% CI)^a^	*p*-Value
**Wasting**	5.6	1,364	4.3	1,360	0.75 (0.53–1.07)	0.12
**Stunting**	25.2	1,365	24.6	1,361	0.96 (0.81–1.15)	0.69
**Underweight**	12.8	1,365	10.9	1,361	0.84 (0.66–1.06)	0.15

a Adjusted for age, sex, and locality.

### The Impact of IPTc on Molecular Markers of Antimalarial Drug Resistance

The frequencies of molecular markers associated with resistance to SP and AQ in the two groups at baseline and postintervention are presented in Table 5. The frequencies of individual and multiple *dhfr* and *dhps* mutations in the placebo group were similar in pre- and postintervention periods. The frequencies of all individual *dhfr and dhps* and of the triple *dhfr* (51, 59, 108) and quadruple *dhfr* (51, 59, 108) *+ dhps* 437 mutations were higher in the intervention than in the control group at the end of the surveillance period and, for the *dhfr* 59, dhps 437, triple and quadruple mutations, differences between groups were statistically significant. Frequencies of the *pfcrt* 76 and *pfmdr1* 86 did not change significantly over time and were similar postintervention in the intervention and control groups.

**Table 5:** Frequencies of molecular markers of resistance to SP and AQ at baseline and at the end of the intervention period in intervention and control arms.

Molecular Markers	Baseline		Postintervention					Baseline Versus Overall Postintervention *p*-Value
			IPTc		Placebo			
	*n*	Percent Mutant	*n*	Percent Mutant	*n*	Percent Mutant	*p*-Value	
DHFR 51	48	62.5	78	75.6	148	66.2	0.144	0.35
DHFR 59	48	60.4	78	76.9	148	59.5	0.009	0.50
DHFR 108	41	78.0	76	78.9	139	71.2	0.217	0.58
DHPS 437	47	38.3	83	67.5	165	43.6	<0.001	0.09
DHPS 540	45	0	82	7.3	165	3.6	0.205	0.82
Triple DHFR mutations	41	58.5	76	69.7	139	54.0	0.024	0.90
Quadruple mutants (triple DHFR + DHPS 437)	41	22.0	75	53.3	139	28.1	< 0.001	0.07
Pfcrt-76	46	80.4	79	84.8	156	75.0	0.085	0.25
Pfmdr1-86	46	45.6	76	36.8	156	34.6	0.739.	0.19

*n* =  number of participants with parasitaemia at blood smear tested.

### The Impact of IPTc on Hospital Admissions and Death

Hospital admissions and deaths that occurred during the study period are listed in Table 6. 19 hospital admissions of at least 24 h were recorded; nine of these were recorded in children in the control arm and ten in children in the intervention arm. The incidence rates of hospital admissions per child/year were 0.0225 episodes in the control group versus 0.0251 in the intervention arm (*p* = 0.81). There were five deaths, two in the control arm and three in the intervention arm. Two of the five deaths were due to malaria (one in each group). Both occurred during hospitalisation while the remaining three deaths occurred at home. On the basis of the results of a verbal autopsy, these deaths were thought to be due to poisoning by traditional medicines, meningitis and anaemia, and secondary bleeding following a circumcision, respectively.

**Table 6:** Hospital admissions and deaths by treatment arms.

Numbering	Treatment Arm	Date	Cause	Outcome
**Hospital admission**				
1	Placebo	11/8/2008	Severe malaria	Recovered
2	Placebo	10/25/2008	Severe anaemia	Recovered
3	Placebo	9/27/2008	Severe malaria	Recovered
4	Placebo	11/9/2008	Severe malaria	Recovered
5	Placebo	12/7/2008	Severe malaria	Recovered
6	Placebo	9/18/2008	Severe malaria	Recovered
7	Placebo	9/16/2008	Severe malaria	Death
8	Placebo	9/18/2008	Severe malaria	Recovered
9	Placebo	11/16/2008	Severe malaria	Recovered
10	IPTc	11/23/2008	Gastro-enteritis	Recovered
11	IPTc	11/5/2008	Severe malaria	Death
12	IPTc	12/2/2008	Respiratory infection	Recovered
13	IPTc	10/11/2008	Gastro-enteritis	Recovered
14	IPTc	8/13/2008	Severe anaemia	Recovered
15	IPTc	9/21/2008	Asthma	Recovered
16	IPTc	12/3/2008	Severe malaria	Recovered
17	IPTc	11/11/2008	Respiratory infection	Recovered
18	IPTc	11/4/2008	Febrile convulsions	Recovered
19	IPTc	9/19/2008	Respiratory infection	Recovered
**Deaths out of hospital** a				
1	Placebo	11/08/08	Intoxication to traditional medicines	—
2	IPTc	09/11/08	Meningitis	—
3	IPTc	09/17/08	Anaemia secondary to circumcision	—

a Does not include death occurred following hospital admissions listed above.

### Safety and Tolerability

There was no serious adverse event related to the study drugs. The frequencies of adverse events following the administration of IPTc with SP+AQ or placebo, using active surveillance are summarized in Table 7. The frequencies of adverse events were similar between the control and intervention arms. However, there was a tendency toward a higher frequency of vomiting and of loss of appetite in the intervention arm compared to the control arm (4.0% versus 1.9%, *p* = 0.06 for vomiting and 1.9% versus 0.8%, *p* = 0.08 for loss of appetite). Proportions of children with skin rash and itching on at least at one occasion were similar between the two arms. Four participants in the intervention arm were withdrawn from the study because of reactions to study drug versus none in the control arm. Two of these children had a documented skin rash at physical examination (one after the first dose of IPT and the other after the second dose of IPT) and these were assessed as being related to study drugs. Both were moderate in intensity, did not involve bullous eruptions, and resolved within 2 d. The parent of the third participant reported itching. Physical examination was normal but the child was withdrawn from the study on precautionary grounds. The fourth participant had an acute respiratory infection at the time of administration of the first dose of IPT. No adverse event was recorded at the time of routine surveillance but the parents requested withdrawal of their child from the study at the time of the second round of IPTc.

**Table 7:** Proportions of children with adverse events on at least one occasion during three rounds of IPTc treatment using the active surveillance.

Adverse Events	IPTc	Placebo	ORs (95% CI)	*p*-Value
	Percent (*n/N*)	Percent (*n/N*)		
Fever	10.1 (69/686)	9.9 (66/669)	1.02 (0.72–1.46)	0.91
Vomiting	4.0 (19/475)	1.9 (9/473)	2.1 (0.96–4.80)	0.06
Drowsiness	0.1 (1/686)	0 (0/669)	—	—
Itching	1.0 (7/686)	0.6 (4/667)	1.7 (0.50–5.86)	0.39
Diarrhoea	6.7 (46/686)	4.6 (31/669)	1.48 (0.92–2.36)	0.10
Skin rash	0.3 (2/686)	0.8 (5/668)	0.39 (0.7–2.0)	0.26
Coughing	8.2 (56/686)	6.0 (40/631)	1.40 (0.92–2.13)	0.12
Loss of appetite	1.9 (13/686)	0.8 (5/668)	2.56 (0.90–7.22)	0.08
Jaundice	0 (0/686)	0.1 (1/667)	—	—

## Discussion

This study has shown that three doses of IPTc with SP+Q given at monthly intervals during the peak transmission season reduced the incidence of uncomplicated and severe malaria by 80% in children 3–59 mo of age who slept under an ITN in three localities in Mali despite the difference in ITN use at baseline. This level of protective efficacy is similar to that reported in a previous trial conducted in an area of Senegal with a coverage of ITNs of less than 1% [7], suggesting that the relative efficacy of IPTc is not reduced by the use of an ITN at the time of the intervention. Two studies have shown that in pregnant women, IPT adds little benefit to the protection afforded by an ITN, at least in multigravidae [18],[19]. This finding is not the case for IPTc in children, as the strategy remained highly efficacious even when deployed in a community with a high usage of ITNs.

Despite the large difference in background incidence of malaria in the three sites, suggesting high variability in transmission intensity, the protective efficacy of IPTc against clinical malaria was high and similar between the three sites. This suggests that similar efficacies of IPTc against clinical malaria can be expected in areas with different transmission intensities and baseline ITN coverage. Surprisingly, Siby and Ouelessebougou, which had a low EIR (less than ten infective bites per person/season), had a higher malaria attack rate than Djoliba, which had a higher EIR (37 infective bites per person/season). High malaria infection and attack rates have been reported previously in the context of a low EIR (3.5 infective bites per person/season) in Mali [20], and similar malaria incidence rates were found in children aged 0–5 y in two areas despite a more than 10-fold difference in EIR [21]. However, these apparently anomalous results could have also been due to imprecision in the determination of the EIR, which can vary markedly with time and space or to a difference in the efficiency of transmission. Early detection and treatment of malaria cases is known to reduce hospital admission and deaths due to malaria [22],[23]. Early detection and prompt treatment was available in our carefully controlled study and the protective effect of IPTc on severe malaria or death might be more marked than we observed if IPTc was deployed in a community that did not have such ready access to health care. Parasite prevalence, as assessed by weekly surveys during the intervention period was reduced by 85% in children who received IPTc, but this difference dropped to 46% at the end of the intervention period suggesting that the prophylactic effect of the last dose of SP+AQ had begun to decline 6 wk after administration, as has been found in studies of IPTi [24].

We observed a 47% reduction in the proportion of children with moderately severe anaemia (Hb <8 g/dl) as a result of administration of IPTc. This impact on anaemia is consistent with the reduction of 45% in incidence of anaemia observed when AS+AQ was given at monthly intervals over 6 mo in Ghana, although in the Ghanaian study there was no difference in the proportion of children with anaemia at the end of the 6-mo intervention [9]. We did not detect any difference between the intervention and control arms in wasting, stunting, or under weight. This finding is consistent with a previous study in Senegal [25], which did not find evidence of an impact of IPTc on wasting, stunting, or being under weight at the end of the transmission season but only on triceps and subscapular skinfold, indicators that were not assessed in our study. However, in line with the Senegalese study, we found an increase in weight gain in the IPTc arm compared to the control arm during the course of the intervention period. More marked effects on nutritional measurements were found during a parallel study conducted in Burkina Faso [26], perhaps because the force of infection was higher in the Burkina Faso than in the Mali study areas and malaria, thus, a more important contributor to impairment of weight gain in the Burkina Faso than in the Mali study areas.

SP+AQ was chosen as the drug combination for use in the trial on the basis of the results of previous studies that had shown this to be an effective combination for IPTc. This drug combination was generally well tolerated and no serious adverse event attributable to the study drugs was reported. The proportions of children with mild-to-moderate adverse events using active surveillance were not significantly different between the two arms, although there was a trend towards a higher frequency of vomiting and loss of appetite in the intervention group. In the parallel study in Burkina using the same drugs, a higher frequency of vomiting was found in the intervention arm [26]. However, even in the placebo group the frequency of vomiting was higher than in this study, suggesting a difference in the way in which minor side effects were solicited in the two study areas. Cisse et al. [7] reported a modest increase in vomiting in children who took SP+AS compared to those who took placebo in Senegal, while Kweku et al. [9] found no difference in incidence of these adverse events between IPTc intervention and control arms when using SP or AQ. Four withdrawals in the intervention arm were reported to be due to reactions to study drugs. In two cases, the presence of a skin rash was confirmed, another child had itching, and the final withdrawal followed the occurrence of an acute respiratory tract infection at the time of administration of the first round of IPTc. It is possible that this event was considered by the parents as a reaction to the study drugs. The safety of SP and AQ has been a concern in relation to their use for IPTc [27]–[30]. However, there is a growing body of evidence from studies in the last few years [4],[6],[7],[9],[26],[30] that these drugs are safe when used for IPT in pregnant women, infants, or children, and no safety concerns have arisen following the use of SP+AQ for IPTc on a large scale in Senegal.

The efficacy of IPTc against clinical malaria has now been demonstrated in a number of studies, including the current trial and a parallel one conducted in Burkina Faso [26]. Is the evidence now strong enough to support the introduction of IPTc into countries with seasonal malaria transmission? Evidence from studies of IPT in infants [31],[32] suggests that prophylaxis is the key protective mechanism of IPT and that long-acting drugs are needed for effective IPTc. Currently, the SP+AQ combination meets this requirement in West Africa where both of these drugs are still reasonably effective as has been shown to be the case in the study area (Text S6). Studies conducted in Senegal and in Ghana [8],[30] have compared different drug combination and regimens and shown that currently SP+AQ at monthly intervals is the best combination. However, the continuing efficacy of SP cannot be guaranteed and alternative regimens for IPTc will be required in the future, which might include the long-acting drug piperaquine.

Unlike the case of IPT in pregnant women and infants, IPT in children has no established delivery system, raising concerns as to whether it could be implemented as a control measure. However, studies conducted in Ghana and The Gambia have shown that high coverage with IPTc can be obtained using community health workers [30],[33], and this appears the most promising way of delivering this intervention.

Another concern over the widespread deployment of IPTc is that this will enhance the spread of drug resistance. Therefore, we studied the presence of molecular markers associated with resistance before and after the intervention in children in the intervention or control group. The *dhfr 59* and *dhps 437* mutations associated with pyrimethamine and sulphadoxine resistance, respectively, were found significantly more frequently at the end of the malaria transmission season in parasites obtained from children in the intervention group than in those obtained from children in the control group, and this led to higher frequencies of the triple *dhfr* mutants and the quadruple mutant (triple *dhfr + dhps 437*) associated with significant resistance to SP in children who had received IPTc. This increase in the frequency of these mutations is consistent with a previous report in Senegal [7]. As in Senegal, the number of children in the intervention group carrying a resistant parasite was less than in children in the control group because of the substantial reduction in the overall prevalence of parasitaemia. Although IPTc may have contributed to the increase in frequency of some of resistant markers in this and other studies, the true impact on the resistance of SP and AQ remains to be established. Despite a prevalence of quadruple mutants of about 37%, the SP+AQ combination was highly effective in clearing parasitaemia from children resident in the study area with asymptomatic parasitaemia (Text S6).

As is the case with any successful malaria intervention, administration of IPTc to children during several, successive malaria transmission seasons could interfere with the development of naturally acquired immunity, raising concerns that there would be an increased period of risk (rebound malaria) during the period immediately after the intervention was stopped if exposure levels remained high. The risk of malaria for children in this trial in the year after the intervention was stopped has been studied and the results are currently being analysed. However, several years of administration would be needed to define the degree to which acquisition of natural immunity would be impaired. It is very unlikely that this would outbalance the substantial gains made during the period when the drug was given.

Our study has several strengths. First, the double-blind, randomised controlled design prevented a number of biases in the selection assignment of the participants to the two arms as well as in assessing the outcomes. A second strength is that this is the largest IPTc efficacy trial done so far, providing a more precise estimation of the outcomes measured. Third, the trial was conducted in three localities with different malaria incidence rates, allowing the efficacy of this strategy under different levels of malaria transmission to be assessed. The design would have been stronger if a factorial design had been used to assess the individual and combined impact of IPTc and ITN, but such a trial would be unethical as the efficacy of ITN is already established [3] and use of ITNs is policy in Mali. Other potential limitations of the study include the duration of evaluation, which focused only on about 15 wk of follow-up during the malaria transmission season. However, it is well established that the in the Sahel region of Mali, 85%–90% of clinical malaria cases occur during the period of August to November, and efficacy of this strategy remained high in a previous, smaller study when efficacy was computed over 12 mo period [6],[34].

In summary, IPTc given during the malaria transmission season, provided substantial additional protection against clinical malaria, infection with malaria, and anaemia to that provided by ITNs. IPTc with SP+AQ was safe and well tolerated. As the international community moves towards the target of malaria elimination, new malaria control tools will be needed [11]. IPT in children targeting the transmission season appears to be one of the strongest available tools to achieve this goal. Our findings support the need for an early review of whether IPTc can now be recommended as a component of malaria control in areas with seasonal malaria transmission.

## Supporting Information

Text S1 Study protocol: A trial of the combined impact of IPT and ITNs on morbidity from malaria in African children.
(0.19 MB PDF)

Text S2 CONSORT checklist.
(0.22 MB DOC)

Text S3 Entomological investigations.
(0.45 MB PDF)

Text S4 External quality assurance of malaria microscopic diagnosis.
(0.10 MB PDF)

Text S5 Protocol amendment.
(0.11 MB PDF)

Text S6 In vivo efficacy of the SP+AQ combination used for IPTc in the study area.
(0.51 MB PDF)

## Abbreviations

AQ: amodiaquine
AS: artesunate
CI: confidence interval
EIR: entomological inoculation rate
Hb: haemoglobin
IPT: intermittent preventive treatment
IPTc: intermittent preventive treatment of malaria in children
IPTi: intermittent preventive treatment of malaria in infants
ITN: insecticide-treated net
LLIN: long-lasting insecticide-treated net
PE: protective effect
RDT: rapid diagnostic test
SP: sulphadoxine pyrimethamine
