# Supplementary material for: Intermittent Preventive Treatment of Malaria Provides Substantial Protection against Malaria in Children Already Protected by an Insecticide-Treated Bednet in Mali: A Randomised, Double-Blind, Placebo-Controlled Trial
Source: PLoS Med. 2011 Feb 1;8(2):e1000407. doi: 10.1371/journal.pmed.1000407 (PMC3032550; doi:10.1371/journal.pmed.1000407)
Supplement: Text S3 — Entomological investigations. (0.45 MB PDF) [file pmed.1000407.s003.pdf]

SUPPLEMENTARY INFORMATION - ENTOMOLOGICAL INVESTIGATIONS (TEXT, S3)

A surprising feature of the study described in this paper is the high malaria attack rate in children who slept under an ITN and who did not receive IPTc. This may have been due in part to the fact ITNs were given only to children in the trial and not to the whole community. However, a number of other studies were undertaken to explore this finding further.

1. Entomological Inoculation Rate in the study area

Introduction

A small study was undertaken in the three localities Djoliba, Siby and Ouelessebougou in the district of Kati in Mali from August to December 2009 to measure the Entomological Inoculation Rate (EIR)

Methods

Catches were performed in 10 houses on two consecutive nights (from 6:00PM to 6:00AM) per week per village using CDC light traps (J. W. Hock Co, Gainesville, Florida, USA).. Trapping bags were emptied the next morning. Mosquitoes collected were then sorted by species, counted and stored in tubes containing silica gel. Tubes were labelled with the following information: village code, date, the mosquito species and numbers by species. Mosquitoes were transported from the field to the laboratory and stored in boxes at room temperature until processing.

Heads and thoraces of mosquitoes caught in the light traps were tested for *Plasmodium falciparum* circumsporozoite protein (CSP) by ELISA essay. A representative sample was tested when the numbers of mosquitoes collected was too large. The EIR was computed for each study site by multiplying the sporozoite rate (proportion of mosquitoes tested positive for CSP by ELISA) by mean vector man biting rate per month.

Results

A total of 10,022 mosquitoes were collected in the three sites (8,444 in Djoliba, 493 in Siby and 1085 in Ouelessebougou) during the 1287 catches (427 in Djoliba, 426 in Siby and 434 in Ouelessebougou) carried out from August to December 2009. The infection rates were 1.1% (35/3282) in Djoliba, 4.9% (17/344) in Siby and 1.8% (16/879) in Ouelessebougou. The cumulative entomological inoculation rates during this five-month period in the three study localities were 37.3 (95% CI -22.3 -44.4), 9.4% (95% CI 5.4-14.6), and 6.6 (95% CI 3.8 -10.7) infective bites per person in Djoliba, Siby and Ouelessebougou respectively.

Conclusions

The EIR in the study sites of 7-37 infective bites per person per season is compatible with an incidence rate of 1-3 clinical attacks of malaria per season in young children in these sites and consistent with previous reports from Mali [1,2] and thus with an attack rate of 0.5 -1.5 clinical attacks per season in children sleeping under an ITN, as observed in this study.

2. Deltamethrin content of treated nets

Introduction

At the beginning of the study, the family of each study child was provided with an LLIN ((PermaNet® Vestergaard) to be used by that child during the course of the study and with instructions on how to use it. To confirm that the ITNs used in the IPTc study had retained chemical activity and were thus likely to be effective, an analysis was made of the deltamethrin content of a random sample of nets being used by study children the end of the malaria transmission season.

Methods

Nets from 49 children randomly selected from the cohort of children enrolled into the study were collected in December 2009 and tested for their content of deltamethrin. Removed mosquito nets were replaced by new ones. Two pieces of netting were obtained from each side of the net and from its roof. The deltamethrin was extracted from the netting samples with solvents and the deltamethrin content of the extract determined by high performance liquid chromatography as previously described [3].

Results

Assays were undertaken on 10 samples (2 per side) obtained from each of 49 nets. Deltamethrin was detected in one sample from all the 49 netting samples tested, confirming that study children had been using an ITN but in some cases only in a few pieces of the net (nets washed >5 times and/or very dirty). However, in most cases content remained in the range likely to be associated with protection.

Conclusions

The results confirmed that the nets used in the study had been treated with an appropriate concentration of deltamethrin. Lower concentrations than expected were found in some samples which may have been due to the fact that the nets had been washed several times, as is common practice in the study area.

3. Pyrethroid resistance

Introduction

A possible explanation for the apparent lack of efficacy of efficacy of the ITNs is that anopheline mosquitoes in the study area have become resistant to deltamethrin and studies were carried out to explore this possibility.

Methods

To assess the resistance of the mosquitoes to the pyrethroids, *An gambiae* s.l., collected in the study areas were tested using the method of WHO (1998). A hundred mosquitoes were exposed to filter paper impregnated with pyrethroids (deltamthrin permethrin) or DDT for one hour and morality after 24 hours was measured. Forty mosquitoes randomly selected among those exposed to each of the three pyrethroids in each of the tree sites (120/site) were tested for the *kdr* gene mutations using the methods described by Martinez-Torres et al. [4] and Ranson et al. [5].

Random samples of 10 long lasting insecticide treated net (LLIN) allocated to study children in each village were removed in October –November 2009 and tested for their killing effect on mosquitoes. Fifty mosquitoes were exposed for three minutes to the LLIN to estimate mosquito knock-down time using the WHO standard protocol [6].

Results

Mortality 24 hours after the exposition to deltamethrin for one hour was 100% in all the three sites. The mortality 24 hours after the exposition to permethrin was 100% in Djoliba, 62% in Ouelessebougou and 46% in Siby. The corresponding figures after exposure to DTT were 100%, 100% and 97% respectively. None of the 360 mosquitoes tested carried the *kdr* gene mutation.

The efficacy of the study nets (defined as mosquitoes mortality 24 hours after exposure to the net) was 100% in Djoliba, 100% in Siby and 94% in Ouelessebougou giving and overall efficacy in the area of was 98% l after an average number of washing of 3.2 (95% CI 2.2- 4.1).

Conclusions

The results of this small study suggest that there is no resistance in *Anopheles gambiae* to deltamethrin in the area where the study was done.

References

1. Dicko A, Sagara I, Diemert D, Sogoba M, Niambele MB et al (2007) Year-to-year variation in the age-specific incidence of clinical malaria in two potential vaccine testing sites in Mali with different levels of malaria transmission intensity. Am J Trop Med Hyg 77:1028-33
2. Coulibaly D, Diallo DA, Thera MA, Dicko A, Guindo AB, et al. (2002) Impact of preseason treatment on incidence of falciparum malaria and parasite density at a site for testing malaria vaccines in Bandiagara, Mali. Am J Trop Med Hyg 67:604-10.
3. Yates A, N'Guessan R, Kaur H, Akogbeto M, Rowland M (2005). Evaluation of KO-Tab 1-2-3[R]: a wash resistant 'dip-it-yourself' insecticide formulation for long-lasting treatment of mosquito nets. Malar J 4:52.
4. Martinez-Torres D, Chandre F, Williamson MS, Darriet F, Bergé JB et al. (1998) Devonshire AL, Guillet P, Pasteur N, Pauron D. Molecular characterization of pyrethroid knockdown resistance (kdr) in the major malaria vector Anopheles gambiae s.s. Insect Mol Biol. 7:179-84
5. Ranson H, Jensen B, Vulule JM, Wang X, Hemingway J et al. (2000) Identification of a point mutation in the voltage-gated sodium channel gene of Kenyan Anopheles gambiae associated with resistance to DDT and pyrethroids. Insect Mol Biol 9:491-7.
6. World Health Organisation – Test procedures of insecticide resistance monitoring in malaria vectors, bio-efficacy and persistence of insecticides on treated surfaces. WHO/CDS/CPC/MAL 98.12:12-17.
