# Supplementary material for: Intermittent Preventive Treatment of Malaria Provides Substantial Protection against Malaria in Children Already Protected by an Insecticide-Treated Bednet in Mali: A Randomised, Double-Blind, Placebo-Controlled Trial
Source: PLoS Med. 2011 Feb 1;8(2):e1000407. doi: 10.1371/journal.pmed.1000407 (PMC3032550; doi:10.1371/journal.pmed.1000407)
Supplement: Text S4 — External quality assurance of malaria microscopic diagnosis. (0.10 MB PDF) [file pmed.1000407.s004.pdf]

KENYA MEDICAL RESEARCH INSTITUTE
U.S. ARMY MEDICAL RESEARCH UNIT-KENYA
MALARIA DIAGNOSTICS AND CONTROL CENTRE OF EXCELLENCE (MDCoE)

EXTERNAL QUALITY ASSURANCE OF MALARIA MICROSCOPIC DIAGNOSIS

17 May 2010

Final Malaria Blood Film Concordance Report for LSHTM IPTc/ITNs Project

Background

Following the Quality Assurance/Quality Control (QA/QC) activity carried out on London School of Hygiene and Tropical Medicine (LSHTM) slides from Mali and Burkina Faso in June and July 2009, site results were sent to Malaria Diagnostics Center of Excellence (MDCoE) for comparative analysis. The objective was to determine the concordance between site and MDCoE results in parasite detection (positive/negative), species identification, and parasite quantitation. Results were entered and merged in Microsoft Access. Slides were classified as either positive or negative if two of the initial readers i.e. reader 1 and 2 results agree or any of the initial reader's result agrees with that of the reference reader in case the initial results were discordant. The same criterion was applied in classification of species. Densities were calculated based on parasite counts against white blood cells (WBC) or counts of parasitized red blood cells (RBC) depending on the counts reported. Counts against WBC were converted using World Health Organization recommend count of 8,000 cells per microliter (µL). Counts of parasitized RBC were converted using 4.5×10^6 cells/µL. Densities were considered to be concordant if the difference between the paired results was within 0.5 – 2.0 fold.

Summary of Reported Results by Site and MDCoE

Classification	MRTC	MDCoE	Agreement	CNRFP	MDCoE	Agreement
Negative	239	228	221	176	167	162
Positive	50	51	43	110	119	105
P. falciparum	50	51	43	106	111	98
P. malariae	0	0	0	2	1	1
P. ovale	0	0	0	0	3	0
P. falciparum + P. malariae	0	0	0	2	3	1
P. falciparum + P. ovale	0	0	0	0	1	0
Gametocytes	4	1	1	16	34	2

MRTC Results

Of the 289 slides submitted only 279 reads were paired. 10 of the slide could not be read because of poor quality of the smears. MRTC reads reported 86% (239/279) as negative and 18% (50/279) as positive. Of the positive reads, all were (50/50) classified as pure *P. falciparum* infections. Gametocytes were reported on 1% (4/279) of the positive slides. MDCoE classified 82% (228/279) as negative and 18% (51/279) as positive. Of the positive reads, all were reported as (51/51) as pure *P. falciparum* infections. Presence of gametocytes was reported on 0.4% (1/279) of the positive reads. Of the two sets of paired reads (MRTC/MDCoE), 95% (264/279) of the reads were concordant on negative/positive classification. 84% (221/264) were classified as negative and 16% (43/264) as positive. All the positive concordant results (43/43) were classified as pure *P. falciparum* infections and 0.4% (1/264) reported as having gametocytes. Cohen's Kappa on agreement based on classification of the 279 paired reads was almost perfect (estimated Kappa 0.8186). Comparison of densities could not be done because the site did not provide the method used for generation of submitted densities.

CNRFP Results

Of the 300 slides submitted only 286 reads were paired. 9 slides were not read due to poor quality of the smears, 3 did not have legible slide identification numbers and 2 of the results were not provided by the site. CNRFP readers reported 62% (176/286) as negative and 38% (110/286) as positive. Of the positive reads, 96% (106/110) were classified as pure *P. falciparum* infections, 2% (2/110) as pure *P. malariae* infections and 2% (2/110) as mixed infections of *P. falciparum* and *P. malariae*. Gametocytes were reported on 15% (16/110) of the slides. MDCoE readers reported 58% (167/286) as negative and 42% (119/286) as negative. Of the positive reads, 93% (111/119) were classified as pure *P. falciparum* infections, 0.8% (1/119) as pure *P. malariae* infection, 2.5% (3/119) as pure *P. ovale* infections, 2.5% (3/119) as mixed infections of *P. falciparum* and *P. malariae* and 0.8% (1/119) as a mixed infection of *P. falciparum* and *P. ovale*. Gametocytes were reported on 29% (34/119) of the slides. Of the two sets of paired reads (CNRFP/MDCoE), 93% (267/286) were concordant on negative/positive classification. Of the concordant reads, 61% (62/267) were classified as negative and 39% (105/267) as positive. Of the concordant positive reads, 93% (98/105) were classified as *P. falciparum*, 1% (1/105) as pure *P. malariae* and 1% (1/105) as mixed infection of *P. falciparum* and *P. malariae*. Gametocyte presence was concordant on 12% (12/105) of the slides. Cohen's Kappa on agreement based on classification of the 286 paired reads was almost perfect (estimated Kappa 0.8617). Of the 105 positive results, only 98 reads with asexual parasites were paired and of these, 66% (65/98) of the densities were within 0.5 – 2.0 fold difference.

Discussion

Concordance rates in parasite detection between sites and MDCoE were very high (>90%) suggesting site microscopists were highly accurate in classifying the slides. Discrepancies noted were mostly on very low density slides (< 200 parasite/µL) and on poor quality smears. Site microscopists were proficient in classifying *P. falciparum* infections accurately; however, CNRFP site had problems with non-*falciparum* infections. To overcome this problem, continuous training/assessment on identification of non-*falciparum* species must be established. As a rule of thump, concordance on parasite densities should ≥ 70%. Even though agreement between CNRFP and MDCoE density was lower than the rule of thump, binomial approximation suggested differences between 0.5 – 2.0 fold were significantly higher than differences outside the specified limits. It is important to note that it is not easy to achieve acceptable concordance on slides with parasite densities < 200 parasites/µL. It is also important to note that acceptable concordance cannot be achieved on poorly prepared slides. In conclusion, the exercise was successful based on the concordance rates achieved within the target parameters. However, more emphasis is needed on improving the competence levels of microscopists, the quality of prepared smears, and quality assurance/control mechanisms.

Peter Obare Signature [signature] Date 17 MAY 2010

Technical Coordinator
Malaria Diagnostics and Control Center of Excellence
Kenya Medical Research Institute/US Army Medical Research Unit-Kenya
Email: pobare@wrp-ksm.org
