# Supplementary material for: Intermittent Preventive Treatment of Malaria Provides Substantial Protection against Malaria in Children Already Protected by an Insecticide-Treated Bednet in Mali: A Randomised, Double-Blind, Placebo-Controlled Trial
Source: PLoS Med. 2011 Feb 1;8(2):e1000407. doi: 10.1371/journal.pmed.1000407 (PMC3032550; doi:10.1371/journal.pmed.1000407)
Supplement: Text S5 — Protocol amendment. (0.11 MB PDF) [file pmed.1000407.s005.pdf]

Dear Prof Meade 11.7.08

A trial of the combined effect of intermittent preventive treatment and insecticide treated bednets in reducing morbidity form malaria in African children"

Ethics committee 5275

I am writing to you to request approval of a number of protocol amendments for this trial which was approved by the ethics committee on March 19th 2008.

The following amendments have become necessary because of the unexplained refusal of the Ghana Food and Drug Board (FDB) to allow the trial to go ahead with sulphadoxine and amodiaquine even though provisional ethical approval had been obtained for the study in Ghana using these drugs. We have been advised that any negotiations over having this decision amended would take many months and, because of the seasonality of malaria in Ghana, this would mean delaying the trial for a year which we cannot afford to do. Therefore, with agreement of the sponsors, we have decided to close this site for this part of the project. This has required the following amendments to the protocol

1) Trial sites

The number of sites has been reduced to two (Burkina Faso and Mali) with exclusion of Navrongo Health Research Centre (NHRC) in Ghana.

2) Sample size

The number of children to be enrolled in the trial in Navrongo was split between MRTC Mali and CNRFP, Burkina Faso. The number of children to be enrolled at each of these two sites has now been increased from 2,000 to 3,000 (1,500 per arm) to ensure that the study has adequate power to measure the effect of IPTc on hospital admissions with malaria, when data from the 2 sites are combined. The numbers of children to be enrolled in the weekly and the monthly surveys and for the surveillance of drug resistance have been re-adjusted.

A number of other protocol amendments have been following a meeting of the investigators held in Ouagadougou 9-11th June 2008 and of the first meeting of the DSMB held on June 25th. These amendments, which are all minor, have been made to improve the quality of the study or to increase safety for the study subjects

3) Randomisation

To minimise the risk of "unblinding", the number of blocks has been increased to 5. Randomization will now be done in permuted blocks of 10 (5 blocks per group) instead of 8 as initially stated.

4) Dosage of sulfadoxine-pyrimethamine (SP) and amodiaquine (AQ)

Dosage of drugs is now based on child's weight instead of age due to the difficulty of correctly estimating child's age in rural communities. In the amended protocol, 3 weight groups were constituted (5-9 kg; 10-18 kg and 19 kg and over). Tablets containing the exact daily dose of amodiaquine or sulfadoxine pyrimethamine will be manufactured for each weight group to avoid breaking tablets, which may results in inadequate dosage of drugs.

5) Intermittent preventive treatment administration

The revised version of the protocol specifies that children who miss the first dose of IPTc administration of a given treatment round must be visited within 3 days of the planned date of visits instead of 7 days and the 3 doses of each treatment course must be given within 7 days

after the first dose. The reason for this is to make sure that the interval between IPTc courses is at least 21 days.

6) Safety of SP and AQ

The initial plan was to conduct weekly visits in a random sample of 100 children to monitor adverse events. The protocol has now been amended so that adverse events will be assessed on days 0, 1 and 2 of IPTc administration and the day after the last dose of IPTc administration. Adverse events that may occur after day 3 post IPTc will be recorded on day 0 of the next round of IPTc administration. This option provides the opportunity for monitoring adverse events in all study children.

7) Baseline survey of markers of resistance to SP and AQ

A random sample of children from the study villages' census lists will be selected for the baseline survey of the prevalence of markers of resistance to SP and AQ instead of a sample of children from neighbouring villages as stated in the previous version of the protocol.

8) Surveillance of malaria morbidity

It was planned indicated that filter paper samples will be taken from sick children to assess the prevalence of markers of resistance to SP and AQ, but it has now been agreed not to take filter paper samples from sick children because the value of these samples to understand the effect of IPTc on drug resistance markers is questionable.

9) End of malaria transmission season cross-sectional survey

The previous version of the protocol indicates that weight and height were the anthropometric parameters to be measured during the survey. After discussion with an anthropologist it was suggested to measure child's arm circumference during this survey. Now the protocol is amended that mid arm circumference will also be measured at baseline and during the end of malaria transmission season survey.

10) Slide reading

The previous version of the protocol states that 100 high power fields were to be examined before a slide is declared negative. According to the revised protocol all fields in a slide must be examined before a blood smear is declared negative for malaria parasites.

The amended protocol and a document tracking modifications are made are attached.

I hope that it will be possible to approve these changes which are all minor with the exception of dropping one study site.

Please let us know if you need any more information

Yours sincerely,

Brian Greenwood

Brian Greenwood
Principal Investigator
