# Supplementary material for: Intermittent Preventive Treatment of Malaria Provides Substantial Protection against Malaria in Children Already Protected by an Insecticide-Treated Bednet in Mali: A Randomised, Double-Blind, Placebo-Controlled Trial
Source: PLoS Med. 2011 Feb 1;8(2):e1000407. doi: 10.1371/journal.pmed.1000407 (PMC3032550; doi:10.1371/journal.pmed.1000407)
Supplement: Text S6 — In vivo efficacy of the SP+AQ combination used for IPTc in the study area. (0.51 MB PDF) [file pmed.1000407.s006.pdf]

SUPPORTING INFORMATION – SP+AQ EFFICACY (TEXT, S6)

In vivo efficacy of the sulphadoxine/pyrimethamine + amodiaquine combination used for IPTc in the study area.

Introduction

In order to confirm the ability of the sulphadoxine/pyrimethamine (SP) + amodiaquine (AQ) combination used in the IPTc study to clear malaria parasitaemia in asymptomatic children, a small observational study was undertaken in children aged 1-6 years with proven *Plasmodium falciparum* parasitaemia but no symptoms who were resident in the study area in 2009, the year after the IPTc intervention had been conducted.

Methods

In November/December 2009, available children enrolled in the main IPTc efficacy study were tested for malaria parasite using a Rapid Diagnostic Test (Optimal®). Blood films obtained from the children were examined for asexual malaria parasitemia if the Rapid Diagnostic Test is positive. A total of 247 assymptmatic children with asexual P. falciparum parasitaemia at blood smear were enrolled in this sub-study and treated with SP + AQ. Doses of SP and AQ were given on the basis of weight as in the IPTc study. SP was given once at the doses of 175/8.75 mg, 350/17.5 mg and 550/26.25 mg to children who weighed 5-9 kg, 10 – 18 kg and 19 kg and above. AQ was given over three days at the daily dose of 70 mg, 140 mg and 220 mg to children who weighed 5- 9 kg, 10-18 kg and 19 or > kg, respectively. Children were followed-up for 28 days with clinical examination at days 0, 1, 2, 7, 14, 21, and 28. Blood smears and blood blotted onto filter paper samples were collected at days 0, 7, 14, 21 and 28 and at any unscheduled visit for fever or a history of fever. Thick blood films were air dried, stained with Giemsa and examined for malaria parasites by two well-trained technicians. One hundred high power fields were counted before a film was declared negative. Parasite density was determined by counting the number of parasites present per white blood cell (WBC) on a thick smear and assuming a WBC count of 8,000 per µl. In the case of a discrepancy (positive/negative or a difference in parasite density greater than 30%), a third reading was done. The median parasite density of two or three readings was used. Re-infections were differentiated from recrudescences on the basis of the detection of different msp 1 and msp 2 alleles in paired samples as previously described[1, 2].

Results

Of the one hundred forty seven children with asymptomatic malaria were enrolled and treated, one was excluded on day 1 because of allergy to study drugs and the remainder received the complete

course of SP+AQ. Two subjects were treated with Coartem (on days 7 and 9 respectively) based on the occurrence of clinical features suggestive of malaria and positive RDT although no asexual malaria parasites were seen on a subsequent blood smear. Blood smears for 27 subjects at day 7 were altered and not readable. Three children had a positive blood film for P. falciparum on day 28. However, all three were classified as reinfections by PCR so that the 28 days PCR corrected efficacy of SP+AQ in clearing *P falciparum* in asymptomatic children was 100% (144/144).

Conclusion

The results of this small observational study confirm the *in vivo* efficacy of the SP + AQ combination in clearing malaria parasitaemia from asymptomatic children in the study area. As noted in previous studies of intermittent preventive treatment in pregnant women and in infants, drugs or drug combination may retain high efficacy in clearing low density parasitamemia from asymptomatic subjects after they have lost some of their efficacy in treating clinical infections in young children.

References

1. Ranford-Cartwright LC, Taylor J, Umasunthar T, Taylor LH, Babiker HA et al. (1997) Molecular analysis of recrudescent parasites in a Plasmodium falciparum drug efficacy trial in Gabon. Trans R Soc Trop Med Hyg 91:719-24
2. Sagara I, Diallo A, Kone M, Coulibaly M, Diawara SI et al. (2008) A randomized trial of artesunate-mefloquine versus artemether-lumefantrine for treatment of uncomplicated Plasmodium falciparum malaria in Mali. Am J Trop Med Hyg 79:655-6
